# Cardiovascular risk factors and cognitive ability across eight decades of life: a cross‐sectional analysis of the Understanding Society UK Household Longitudinal Study

**DOI:** 10.1002/alz70860_101505

**Published:** 2025-12-23

**Authors:** Scott T Chiesa, Kristine B Walhovd, Meena Kumari, Alun D Hughes

**Affiliations:** ^1^ UCL Unit for Lifelong Health and Ageing, London, England, United Kingdom; ^2^ University of Oslo, Oslo, Norway; ^3^ University of Essex, Colchester, England, United Kingdom; ^4^ UCL Unit for Lifelong Health & Ageing, London, England, United Kingdom

## Abstract

**Background:**

Cardiovascular risk factor exposures in midlife have repeatedly been linked to cognitive impairment and dementia. How early in the lifespan these associations emerge, however, and whether they remain stable over time, remains poorly studied. This study aimed to characterize lifespan associations between cardiovascular risk factors and cognitive ability in a cross‐sectional study of up to 34,684 participants aged 20–100 years participating in Understanding Society: The UK Household Longitudinal Study.

**Method:**

Multivariable regression analyses with interaction terms were tested across each decade of age to cross‐sectionally characterise associations between optimal and suboptimal levels of eight risk factors (BMI, HbA1c, SBP and DBP, non‐HDL and HDL cholesterol, smoking status, and sleep duration) and a latent measure of cognitive ability underlying five cognitive domains (immediate/delayed verbal declarative memory, working memory/concentration, semantic fluency, and reasoning). All analyses were adjusted for sex and highest attained education level. Inverse‐probability weighting was used to ensure results were representative of the UK population.

**Result:**

Mean cognitive ability was stable in the cohort in young adulthood, showed moderate decline across midlife, and accelerated decline from age 70 onwards. Higher levels of HbA1c, lower levels of HDLc, smoking, and poor sleep were found to consistently associate with lower levels of cognitive ability across virtually the entire lifespan (main effect *p* <0.001 for all risk factors). Higher BMI – but not blood pressure – was found to associate with lower cognitive ability during midlife alone. The majority of these associations were either lost or reversed in the oldest old, albeit with less precise estimates at this age due to lower participant numbers.

**Conclusion:**

Commonly observed associations linking midlife cardiovascular risk factors to cognitive impairment may in part represent established differences persisting from the earliest decades of life. Future work investigating the causality and directionality of these relationships is warranted.

